# The Impact of COVID-19 on the Interrelation of Physical Activity, Screen Time and Health-Related Quality of Life in Children and Adolescents in Germany: Results of the Motorik-Modul Study

**DOI:** 10.3390/children8020098

**Published:** 2021-02-02

**Authors:** Kathrin Wunsch, Carina Nigg, Claudia Niessner, Steffen C. E. Schmidt, Doris Oriwol, Anke Hanssen-Doose, Alexander Burchartz, Ana Eichsteller, Simon Kolb, Annette Worth, Alexander Woll

**Affiliations:** 1 Institute of Sports and Sports Science, Karlsruhe Institute of Technology, 76131 Karlsruhe, Germany; carina.mnich9@kit.edu (C.N.); steffen.schmidt@kit.edu (S.C.E.S.); doris.oriwol@kit.edu (D.O.); alexander.burchartz@kit.edu (A.B.); ana.eichsteller@kit.edu (A.E.); simon.kolb@kit.edu (S.K.); Alexander.woll@kit.edu (A.W.); 2 Institute of Movement and Sport, University of Education Karlsruhe, 76133 Karlsruhe, Germany; anke.hanssen-doose@ph-karlsruhe.de (A.H.-D.); annette.worth@ph-karlsruhe.de (A.W.)

**Keywords:** KIDSCREEN-10, subjective physical activity assessment, COVID-19, coronavirus, physical activity, screen time, sedentary behavior, quality of life

## Abstract

Reduced physical activity (PA) and prolonged screen time (ST) negatively influence health-related quality of life (HRQoL), a protective factor against illness and mortality. Studies addressing the relationship between PA, ST, and mental health in youth are scarce, especially in times with high mental health burdens like the COVID-19 pandemic. The purpose of this examination was to investigate whether PA, ST, and HRQoL before COVID-19 predict PA, ST, and HRQoL during the COVID-19 pandemic. Participants from the Motorik-Modul Study (MoMo; *N* = 1711; *M*_age_ = 10.36 (SD = 4.04) years, female = 49.8%; healthy weight = 76.8%) self-reported their PA and ST as well as HRQoL both before and during COVID-19. Relationships of all variables, from before to during COVID-19, were investigated through a path prediction model. Results showed all variables during COVID-19 were predicted by the respective levels before COVID-19, independent of gender and age. Cross-lags revealed a negative influence of before COVID-19 ST on during COVID-19 PA. HRQoL before COVID-19 was positively associated with during COVID-19 PA in children younger than 10 years and females, but not in adolescents and boys. As age- and gender-independent negative influence of before COVID-19 ST on during COVID-19 PA has been detected, health policy may be advised to focus on a general reduction in ST instead of PA enhancement to ensure high PA levels.

## 1. Introduction

On 11 March 2020, the WHO characterized COVID-19, a disease caused by the SARS-CoV-2 virus, as a pandemic [1]. To slow down infection rates, several public health precautions have been adopted worldwide, including extensive physical distancing, isolation policies, and closures of schools, parks, sports facilities, and recreational sites, leading to a restructuring of everyday life. These precautions are assumed to reinforce the current pandemics of global physical inactivity and sedentary behavior [2,3,4], mainly due to school and office closures and suspension of organized sports facilities and activities. Until today, several recommendations have been published to help people counteract negative developments regarding those two behaviors (e.g., [5,6,7,8]), focusing, for example, on outdoor activities or digital home workout activities [9]. Also, researchers warn that those precautions may endanger children’s mental health due to various stressors such as frustration and boredom, lack of in-person contact with classmates, friends, and teachers, inadequate information, lack of personal space at home, and family financial loss [10,11]. Especially screen time (ST) inevitably increased during the pandemic, as children and adolescents were forced to use media for communicational purposes (e.g., meeting online with friends for educational purposes and to prevent isolation) [12,13]. Due to these and other facets of daily life being transferred to an online environment, time spent in physical activity (PA) (e.g., commuting to school and leisure time activities) was attenuated [14]. However, nothing is known yet about how PA, sedentary behavior including ST, and mental health in children and adolescents before and during the COVID-19 pandemic relate to each other. The importance of this topic becomes obvious by looking at the current guidelines for PA recently released by the World Health Organization (WHO; [15]), where sedentary behavior was included in the activity recommendations for the first time.

Based on studies conducted before COVID-19, it is well-known that reduced PA and prolonged sedentary behavior, especially ST, are linked to both negative physical and mental health outcomes in children and adolescents as well as in (later) adulthood [15,16,17,18,19,20]. Some studies indicate that sedentary behavior including ST may be even more important for mental health outcomes than PA (e.g., [4]). For example, a recent systematic review revealed that meeting recommendations for sedentary behavior, operationalized through less than 2 h of recreational ST per day, was associated with more mental health benefits than meeting the PA recommendations [21]. Nonetheless, globally, more than 70% of 1.6 million adolescents failed to achieve sufficient PA in 2016 [22]. Simultaneously, adolescents spend around 6 to 8 h per day in sedentary positions, especially in ST behaviors such as TV viewing, computer use, and gaming [23,24,25], which have presumably increased during the COVID-19 pandemic [26].

Health-related quality of life (HRQoL) generally relates to the individuals’ self-perceived health and includes physiological, psychological, and functional aspects of health and wellbeing [27]. As HRQoL is closely related to physical (e.g., [28]) and mental health status (e.g., [29]), it is a powerful multidimensional construct in the operationalization of perceived health [30]. In children and adolescents, HRQoL contains dimensions concerning physical and psychological wellbeing, experiences, and relations in different environments such as their family, school, and peers [31]. Several studies conducted pre-COVID-19 assessed the relation between PA, ST, and HRQoL in children and adolescents. A systematic review identified 31 (primarily cross-sectional) studies that investigated the relationship between PA and HRQoL or sedentary behavior and HRQoL, showing that higher PA and lower sedentary behavior, commonly assessed through ST, were associated with higher HRQoL [32]. A most recent study revealed self-reported PA to be a positive predictor for almost all facets of HRQoL in a representative German sample of children and adolescents [33]. However, the relationship of both behaviors (PA and ST) with mental health is not entirely clear yet [2]. Pearson and colleagues revealed a negative, but small association between PA and sedentary behavior in young people, suggesting that these behaviors are mostly independent of each other. However, most studies included in their meta-analysis were cross-sectional and thus do not allow any conclusions about prospective or causal relationships [34].

In contrast to before COVID-19, research about relationships between PA, ST, and HRQoL during COVID-19 is scarce. During the lockdown, schools, shops, and sports facilities were legally bound to shut down while the government strongly recommended staying at home whenever possible. Schooling was transferred to homeschooling which posed significant life changes to children and adolescents [11]. Those significant life changes have also impacted the daily life structure of youths, which is assumed to relate to obesogenic behaviors: the structured day hypothesis [35] postulates that people engage more in obesogenic behaviors (e.g., ST) during unstructured days, such as weekend days and holidays [24,25]. Although the unstructured day hypothesis did not include a pandemic situation, it may be applied to the lockdown situation. In the “German School Barometer Study”, a survey including about 1000 teachers during Germany’s first lockdown, 84% stated that they used task sheets for teaching, while only 14% stated that they used video conferences for teaching [36]. The high use of task sheets and the low use of video conferences indicates that the day was less structured through regular online classes, but rather that children and adolescents could choose by themselves when to engage in learning activities. Interestingly, in Germany, the lockdown has resulted in increased PA, which is in contrast to the structured day hypothesis, while increases in leisure ST support the structured day hypothesis [37]. However, in several other studies, decreases in both PA and SB were observed, but those studies commonly investigated relationships between the same behaviors and not across ST and PA behaviors [38,39,40,41,42,43].

Until today, studies examining the effects of COVID-19 on mental health, specifically HRQoL in children and adolescents, are rare. In Germany, the COPSY-study revealed a significant decrease in HRQoL in children, adolescents, and adults [44]. Besides, Rajkumar [45] provided a review of literature on mental health symptoms including anxiety and depression and interventions relevant to the COVID-19 pandemic and revealed 28 articles that examined the general population, healthcare workers, and vulnerable populations. The review only included four original research articles and revealed negative effects of the pandemic on mental health outcomes in different populations [45]. However, as HRQoL is a more comprehensive construct than mental health, those results are difficult to compare [46]. Even less is known about how PA and ST relate to HRQoL during the pandemic. Pieh and colleagues [47] examined cross-sectional associations between PA and HRQoL during the COVID-19 lockdown in Australia. However, only adults were included in this study, revealing positive associations between PA and HRQoL for adults younger than 35 years. Studies addressing the relationship between ST and mental health or HRQoL in children and adolescents are missing.

Taken together, a better understanding of the associations between PA, ST as an indicator of sedentary behavior, and HRQoL as an indicator of mental health during this pandemic among children and adolescents is necessary to understand how lifestyle behaviors prior to a pandemic may be protective for mental health in a pandemic situation. To date, no studies, especially no longitudinal studies, were found that investigated PA, ST, and HRQoL prior to and during the COVID-19 lockdown in children and adolescents. Thus, the current study aimed to examine the direct influence of the COVID-19 lockdown on cross-behavioral associations between PA, ST, and HRQoL in a nationwide children and adolescent sample in Germany by using data of the MoMo Wave 3 [48] as pre-Covid-19 baseline and data collected from the same cohort in April 2020 for within-Covid-19 measures. The following hypotheses are tested using a cross-lagged panel design:

**Hypothesis** **1** **(H1).** *Pre-Covid-19 PA positively predicts HRQoL within-Covid-19*.

**Hypothesis** **2** **(H2).** *Pre-Covid-19 ST negatively predicts HRQoL within-Covid-19*.

**Hypothesis** **3** **(H3).** *Pre-Covid-19 PA negatively predicts ST within-Covid-19*.

**Hypothesis** **4** **(H4).** *Pre-Covid-19 ST negatively predicts PA within-Covid-19*.

Additionally, the impact of HRQoL before Covid-19 on PA and ST during Covid-19 was investigated using an exploratory approach, as we are not aware of any studies that previously explored HRQoL as a predictor of PA and ST in a longitudinal design.

## 2. Materials and Methods

The current manuscript is reported based on STROBE guidelines [49]. The full STROBE checklist can be found in Supplementary Table S1.

### 2.1. Procedures

The study was conducted according to the Declaration of Helsinki. Ethics approval was obtained by the Charité Universitätsmedizin Berlin (Baseline Study) ethics committee, by the University of Konstanz (Wave 1) and the Karlsruhe Institute of Technology (KIT) (Wave 2 and 3, a positive ethics vote was given from on 23 September 2014 by the ethics committee of the KIT). The Federal Commissioner for “data protection” and “freedom of information” and the Federal Office for the Protection of Data were informed about the study and approved it. Children and adolescents participated voluntarily. They provided written informed assent and (parental) consent. Data were derived from children and adolescents aged between 4 and 17 years within the third follow-up of the Motorik-Modul Study (MoMo Wave 3; [48]). MoMo applies a cohort sequence design, which means that in addition to the longitudinal observations of the MoMo participants of the baseline study (2003–2006), at each follow-up, a representative youth sample in Germany is recruited. MoMo Wave 3 started in August 2018 and was planned to be finished in June 2020, but due to the COVID-19 situation and the related lockdown in Germany, the study had to be interrupted in March 2020. Participants who took part in MoMo Wave 3 before the Covid-19 lockdown (referred to as pre-COVID-19) were asked to answer an online questionnaire (recruitment: 04/20/2020–04/30/2020) during the COVID-19 lockdown (referred to as within-COVID-19). The time between the two measurements ranged from 1 to 27 months.

**Figure 1:**
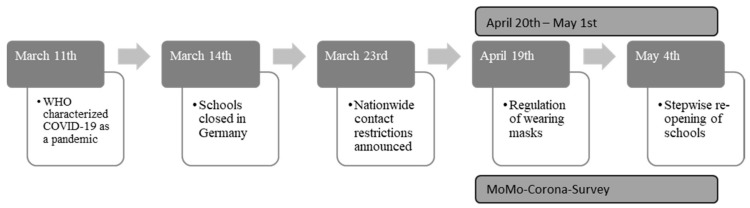
Survey administration schedule regarding COVID-19 restrictions.

Preceding the lockdown, participants were invited to examination rooms within a distance of 15 km from their homes and answered the MoMo physical activity questionnaire (MoMo-PAQ) on laptops. For children younger than 11 years, parents filled in the questionnaire. During COVID-19, every participant who completed the questionnaire of MoMo Wave 3 was again contacted and asked to answer the questionnaire online through a link sent to them via e-mail. The survey administration schedule can be found in Figure 1, embedded in COVID-19 regulations.

### 2.2. Participants

MoMo Wave 3 participants (pre-COVID-19) were selected based on a nationwide multi-stage sampling approach with two evaluation levels to ensure representativeness [50]. First, 167 sampling units were systematically selected from an inventory of German communities which assesses the level of urbanization and geographical distribution [51]. Second, an age-stratified sample based on the official population registers was drawn and invited to the study.

Overall, 2843 participants of MoMo Wave 3 (preliminary response 25.2%) were contacted for the within-COVID-19 assessment, and data from 1711 participants were gathered. Twenty-three e-mails could not be delivered because of incorrect addresses and total longitudinal response of 63.6% was achieved. The reasons for non-participation are unknown due to data protection regulations. For statistical non-responder analyses see results.

### 2.3. Measures

All information was gained using electronic devices, either before the COVID-19 lockdown (pre-COVID-19), or during it (within-COVID-19). Demographic data were only assessed before COVID-19.

#### 2.3.1. Demographics

Demographic data were based on information from MoMo Wave 3 data. The highest educational degree of parents was asked for and parental education was categorized as low, medium, and high according to the CASMIN-classification [52]. Moreover, the height and weight of children and adolescents were measured by trained research staff. Then, the body mass index (BMI) was calculated and participants were assigned as having a healthy weight, being underweight, being overweight, or being obese based on the cut-off points of the International Obesity Task Force (IOTF) [53].

#### 2.3.2. Physical Activity (PA)

Data for PA quantification were derived from the MoMo physical activity questionnaire (MoMo-PAQ), which is sufficiently reliable (test-retest reliability: ICC  =  0.68) and valid [54]; a detailed description and instructions can be found in Schmidt and colleagues [55]. For the current examination, a single item that asked participants for the number of days with at least 60 min PA during a typical week, once for pre-COVID-19, once for within-COVID-19, was used to determine adherence to the WHO guidelines [20].

#### 2.3.3. Screen Time (ST)

ST was measured via self-reported screen-time behaviors. Participants were asked to report the time spent watching TV, playing games on any device, and using the internet for recreational use [56] separately for weekdays and weekends using a 7-point scale including (almost) never, 15 min per day, 30 min per day, 1 h per day, 2 h per day, 3 h per day, and 4 h per day. ST was summed up to gain a total amount of leisure-time media usage in minutes per day.

#### 2.3.4. Health-Related Quality of Life (HRQoL)

To assess adolescents’ subjective health and well-being, the KIDSCREEN-10 index was used [46,57], a short-form of the KIDSCREEN-27. The KIDSCREEN-10 assesses the dimensions of physical and psychological well-being, autonomy and parental relation, peers and social support, and school environment [46]. All questions start with *“Thinking about the last week…”*. Questions include, for example, *“Have you felt fit and well?”* or *“Have you had fun with your friends?”*.

Items were assessed on a five-point Likert-scale ranging from *never* to *always* or from *not at all* to *extremely* and were reversed where necessary according to the manual to ensure higher scores indicate better HRQoL [57]. On a Rasch model basis, a scoring algorithm was used to calculate standardized T-scores scaled with a mean of 50 and a standard deviation of 10 for each dimension to make the interpretation more applicable [58]. Subsequently, the mean T-Score of all KIDSCREEN-10 items was built to obtain an index as a global indicator of overall HRQoL [57]. The KIDSCREEN-10 questionnaire has been repeatedly shown to generate reliable results (Cronbach’s α = 0.82) as well as to have good cross-national validity [46].

### 2.4. Data Analysis

Significance was set to *p* < 0.05 before data analysis. All analyses were conducted using IBM SPSS 26 and AMOS 26. Prior to our analysis, study completers and non-completers were compared using independent sample t-tests and chi-square tests. Additionally, means and standard deviations were calculated for the KIDSCREEN-10 index, self-reported PA, and ST before and during COVID-19, respectively. Descriptive statistics were calculated for the overall sample and stratified by gender and age groups (4–10 years/11–17 years). In the next step, a path panel prediction model with HRQoL, PA, and ST pre- and within-COVID-19 was set up. Path panel models belong to the group of structural equation models and are suitable to examine bidirectional relationships between repeatedly measured variables. This allowed to investigate if relationships occur in both directions and to assess the relative strength of the cross-lagged associations [59,60]. Cross-lagged panel designs have been previously used to investigate similar research questions. In our models, stability coefficients between respective behaviors and cross-lags between different behaviors were added to obtain a comprehensive picture. Full information maximum likelihood (FIML) in AMOS was applied to handle missing data which has been shown to lead to accurate parameter estimates and fit indices with up to 25% missing data [61]. Model fit was evaluated based on the root mean square error of approximation (RMSEA; values ≤ 0.05 indicating a good model fit) and the comparative fit index (CFI; values ≥ 0.90 indicating a good fit and values ≥ 0.95 indicating a very good model fit) [62]. As the models were overfitted when including all cross-lags, bivariate correlations were run between all variables included in the model to examine if a significant linear relationship exists [63]. Only longitudinal relationships that turned out to be significant were included in the model [18]. Due to observed gender and age differences regarding PA and ST, analyses were stratified by gender (boys/girls) and age groups, dividing the sample into participants that were of primary school age or younger (children; 4–10 years) and secondary-school age (adolescents; 11–17 years) before COVID-19. Results are reported as standardized beta coefficients. To account for the effects of outliers of HRQoL, ST, and PA, values >/< than ±2 SD around the mean were put on a maximum level of ±2 SD. Also, multivariate outliers were identified by calculating the Mahalanobis distance [63]. Cases with *p* < 0.001 for the χ2-value were defined as outliers. The models were then re-run with univariate outliers restricted to 2 SD around the mean and multivariate outliers excluded.

## 3. Results

### 3.1. Descriptive Results

Pre-COVID-19, a total of 2843 children and adolescents that would have been eligible for our study participated in the measurement. Within- COVID-19, a total of 1711 children participated, forming the longitudinal sample (*M*_age_ = 10.36 (SD = 4.04) years, female = 49.8%; healthy weight = 76.8%). On average, 11.26 (SD = 5.72) months transpired between the pre- and within-COVID-19 measurements. Regarding age groups, 961 participants were between four and ten years, and 750 children were between ten and 17 years. A more detailed description of the age structure of our sample has been reported elsewhere [37]. In total, 27.0% of the mothers and 31.4% of the fathers of the participating children had a university degree. Sociodemographic differences between study completers and non-completers were observed regarding gender (*p* = 0.049, φ = 0.04), weight status (*p* < 0.001, *V* = 0.09), and parental education (*p* < 0.001, *V* = 0.09). Regarding the study variables, study completers and non-completers differed at baseline regarding PA (*p* = 0.019; *d* = 0.11) and ST (*p* < 0.001, *d* = 0.17). No significant differences for age (*p* = 0.094, *d* = 0.06) and HRQoL (*p* = 0.212; *d* = 0.05) were observed. Detailed information on the differences between study completers and non-completers can be found in Supplementary Table S1. Missing data were 15% for HRQoL pre- COVID-19 and 5% within-COVID-19, 0.01% for PA pre-COVID-19 and no missing data within-COVID-19, and 0.01% for ST pre- COVID-19 and 0.001% within-COVID-19. Detailed results of study-completers vs. non-completers can be found in Supplementary Table S1. Descriptive results of the completers included in the analysis are presented in Table 1.

### 3.2. Cross-Lagged Panel Analyses

As stated in the statistical methods section, including all cross-lags between the variables in the path model led to overfitted models. Thus, bivariate correlations were calculated for the whole sample and the sub-groups of interest. As ST pre- COVID-19 was consistently unrelated to HRQoL within-COVID-19, this path was excluded from the analysis to avoid overfitting of the model.

**Figure 2:**
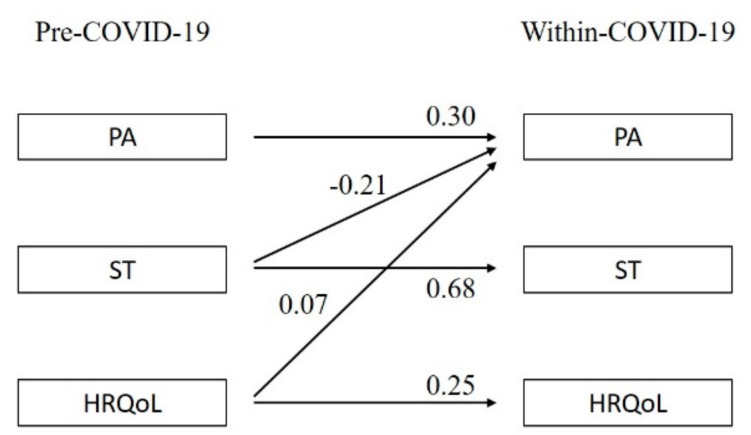
Relationships between PA, ST, and HRQoL pre- and within-COVID-19. Note: PA = physical activity, ST = sedentary time, HRQoL = health-related quality of life. Only significant relationships (*p* < 0.05) are reported. All estimates are standardized estimates.

**Figure 3:**
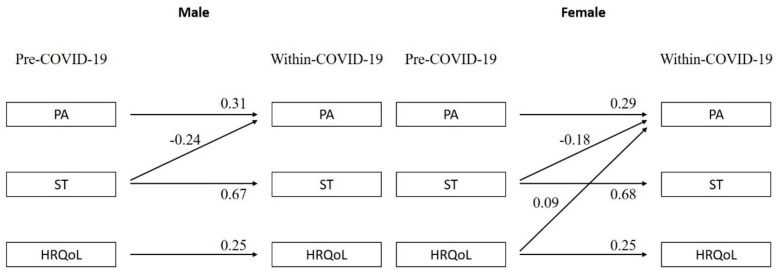
Relationships between PA, ST, and HRQoL pre- and within-COVID-19 stratified by gender (males and females). Note: PA = physical activity, ST = sedentary time, HRQoL = health-related quality of life. Only significant relationships (*p* < 0.05) are reported. All estimates are standardized estimates.

**Figure 4:**
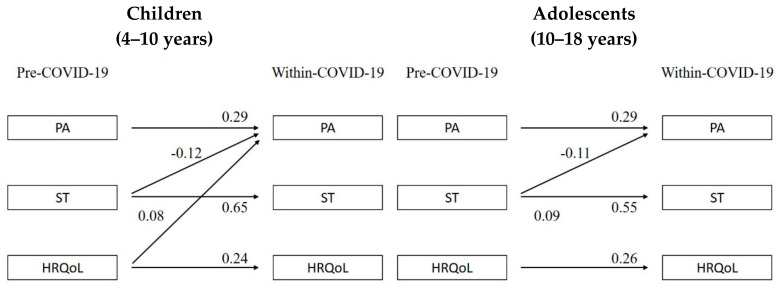
Relationships between PA, ST, and HRQoL pre- and within-COVID-19 stratified by age group (children; 4–10 years/adolescents; 11–17 years). Note: PA = physical activity, ST = sedentary time, HRQoL = health-related quality of life. Only significant relationships (*p* < 0.05) are reported. All estimates are standardized estimates.

All models revealed good fit indices (CFI > 0.95; RMSEA < 0.03). In the following, all estimates are presented as standardized estimates. The strongest relationship between corresponding variables pre- and within-COVID-19 was observed for ST across all models (standardized estimate = 0.55–0.68; *p* < 0.001; see Figure 2, Figure 3 and Figure 4).

The overall model results are presented in Figure 2. Taking all participants together, PA within-COVID-19 was positively predicted by pre-COVID-19 HRQoL (standardized estimate = 0.07; *p* = 0.003) and negatively predicted by pre-COVID-19 ST (standardized estimate = −0.21; *p* < 0.001).

Results for the stratified analysis by gender are presented in Figure 3. For males, ST pre- COVID-19 negatively predicted within-COVID-19 PA (standardized estimate = −0.24; *p* < 0.001), but no statistically significant relationship between HRQoL and PA was observed (*p* = 0.112). For females, PA within-COVID-19 was positively predicted by HRQoL pre-COVID-19 (standardized estimate = 0.09, *p* = 0.007) and negatively by leisure ST pre-COVID-19 (standardized estimate = −0.18, *p* < 0.001; see Figure 2).

Results for the stratified analyses by age group are presented in Figure 4. For children (4–10 years), PA within-COVID-19 was positively predicted by HRQoL (standardized estimate = 0.08; *p* = 0.022) and negatively predicted by pre-COVID-19 ST (standardized estimate = −0.12; *p* < 0.001). For adolescents (11–17 years), PA was negatively predicted by pre-COVID-19 ST (standardized estimate = −0.11; *p* < 0.001), but not HRQoL (*p* = 0.373).

Neither PA nor ST pre-COVID-19 predicted HRQoL within-COVID-19 (all *p*’s > 0.05). Re-run analyses restricting outliers to ±2 SD around the mean (*N* = 294) and excluding multivariate outliers (*N* = 6) did not change the results.

## 4. Discussion

This study aimed to analyze cross-behavioral relationships between PA, ST, and HRQoL pre- and within-COVID-19. Results showed that within-COVID-19 variables were predicted by the same variables pre-COVID-19, independent of gender and age. Cross-lags revealed a negative influence of pre-COVID-19 ST on within-COVID-19 PA, also independent of gender and age, thus confirming Hypothesis 4. None of the other Hypotheses (1–3) were confirmed. Exploratory analyses revealed that HRQoL pre-COVID-19 was positively associated with within-COVID-19 PA in children younger than 10 years and females, but not in adolescents and boys.

The interrelation between the same variables pre-COVID-19 and within-COVID-19 is in line with current literature, even if there are only a few studies to date assessing changes in variables due to the pandemic restrictions (e.g., [2,10,38,40,41,42,43,45,64,65]).

Actual observed HRQoL levels decreased in both age groups and were rather low compared to European norms [57]. Compared to individual norm data of German children and adolescents aged 8 to 18 years [57], the current sample scored quite low regarding their HRQoL, as has also been shown elsewhere [33]. Prior to the COVID-19 lockdown, mean T-Scores were 44 points at the 27th percentile. During the lockdown, scores decreased to 41 points, reflecting the 18th percentile. The demonstrated decrease in HRQoL within-COVID-19 is in line with the COPSY-study led by the University Medical Center Hamburg-Eppendorf (UKE) [44] with *N* = 1040 German children and adolescents aged 11 to 17 years and *N* = 1586 parents. A reason for this general decrease may be lockdown-related changes in children’s and adolescent’s everyday life, including lack of personal space at home, family financial loss, and lack of in-person contact with classmates, friends, and teachers [10,11].

Levels of PA and ST increased within-COVID-19 compared to levels pre-COVID-19, whereas pre-COVID-19 PA levels are comparable to the German state in 2018 [66,67]. A detailed discussion of those results can be found elsewhere [37].

Across all models, a consistent relationship was observed between ST and PA. Children and adolescents with more leisure ST pre-COVID-19 engaged in less PA within-COVID-19. This result is supported by a meta-analysis that was conducted prior to the COVID-19 outbreak [34] and is in line with the unstructured day hypothesis which states that children are more susceptible to obesogenic behaviors on unstructured days [35]. Simultaneously, PA was not prospectively associated with ST. Thus, results indicate lower ST to be a protective factor for PA engagement. However, high PA levels pre-COVID-19 did not prevent ST during the lockdown situation [18]. At this point, studies on compensation behaviors for PA are missing.

The exploratory analysis revealed a positive effect of HRQoL pre-COVID-19 on PA within-COVID-19, especially in younger children and in females. Those results are in line with former studies [18,68,69], revealing a positive relation between HRQoL and PA. For example, Nigg et al. [18] showed that mental and psychosocial health indicators predicted PA from early to later childhood and mostly in females, while there were few inverse relationships. A reason for this could be that children with higher HRQoL scores have more resources in different dimensions, e.g., cognitive and motivational resources, social resources (e.g., parental support), material resources (e.g., sports equipment) that allow them to stay active during a lockdown. The fact that this effect was not present in adolescents may be explained by social restructuring, which commonly takes place during puberty. In this phase, HRQoL may be degraded through other psychosocial factors that might be more prominent indicators here, like rumination, for example. Moreover, the school environment is different for children and adolescents, providing another potential reason due to higher stress burdens related to academic achievement in adolescents.

Within-COVID-19 HRQoL was neither predicted by PA nor ST pre-COVID-19, revealing that habitual high PA pre-COVID-19 was not able to diminish negative mental health outcomes during the pandemic. This finding is somewhat counterintuitive, as other COVID-19-related research found, for example, a significant positive correlation between the variation of PA and mental well-being, suggesting that reduction in total PA is related to a worse status of psychological well-being [70]. A reason for this could be that prior to the pandemic, factors other than lifestyle behaviors may be more important to maintain good mental health than lifestyle behaviors for children and adolescents (e.g., adverse childhood experiences [71]).

To the best of our knowledge, this is the first longitudinal study to examine the direct influence of the COVID-19 lockdown on cross-behavioral relationships between PA, ST, and HRQoL in a nationwide children and adolescent sample in Germany. The use of a multidimensional, validated measure of HRQoL was facilitated to account for national differences and made results comparable to (European) norms. Another strength of this study is its cross-lagged analyses, which allowed us to examine prospective relationships between all variables of interest. Besides, this study covered a wide age range, from kindergarten to late adolescence, and external validity can be classified as high based on the representative nature of MoMo.

However, some limitations also need to be considered. The period between the two assessments had a large range. However, we calculated separate models for baseline assessments made in 2018, 2019, and 2020, which did not change the results. Study completers significantly differed regarding gender, the socioecological status of parents, and time spent with at least 60 min PA as well as ST, which may have biased results and needs to be taken into account when interpreting results. All data were gathered using self-report, which are more likely to be influenced by social desirability than device-based measures and therefore need to be interpreted carefully [72]. Data of children younger than 11 years were provided by parents. Additionally, the KIDSCREEN may not be specific enough to assess HRQoL in a pandemic situation as especially the school environment (which is addressed in the HRQoL questionnaire) has dramatically changed during COVID-19 compared to the situation before the pandemic. However, this tool was chosen to use a validated tool and to allow for comparisons of pre-COVID-19 and during-COVID-19 data. No assumptions can be drawn based on our data as to whether homeschooling had any influence on ST levels. In this vein, it also needs to be noted that pre- and during-COVID-19 measures lay up to eight months apart and most participants grew one year older in the meantime. Especially at young ages, age-related influence and rapid changes within the sociocultural environment of children and adolescents should be considered when interpreting results.

## 5. Conclusions

The results of the current study revealed that any actions to improve HRQoL, especially in children aged 4 to 10 years and females, should be promoted to build resilience for challenging situations like the COVID-19 pandemic. Due to the negative association between pre-COVID-19 ST and within-COVID-19 PA, health policy may be advised to implement measures that lead to a reduction in ST. From a public health perspective, a better understanding of how healthy lifestyles, such as increased PA, relate to HRQoL may help to inform policy intended to incentivize PA in the general population.

Future studies should investigate relationships between PA, ST, and HRQoL in experimental designs and investigate prospective relationships between the three constructs when COVID-19 is not present anymore. Studies should also use device-based measures of PA and HRQoL in children and adolescents and future studies should expand upon the dose-response relationship between PA and HRQoL using continuous measures of both.

## Figures and Tables

**Table 1:** Descriptive results for the study sample (*N* = 1711) pre- and within-COVID-19, stratified by age and gender.

	Pre-COVID-19									Within-COVID-19		
	Male			Female			Male			Female		
	*N*	M	SD	*N*	M	SD	*N*	M	SD	*N*	M	SD
Children (4–10 years, N = 961 female)	514			447								
PA (days/week with at least 60 min of PA)	513	4.74	1.73	447	4.62	1.82	515	5.39	1.88	449	5.27	1.77
ST (min/day)	510	76.65	67.31	442	74.61	74.31	514	143.73	104.04	449	124.86	97.70
HRQoL (KIDSCREEN-10 T-Score)	406	44.89	3.92	346	45.49	4.88	472	40.68	4.33	414	41.27	4.34
Adolescents (11–17 years, N = 750)	345			405								
PA (days/week with at least 60 min of PA)	343	3.90	1.66	399	3.55	1.66	344	4.08	2.02	403	3.96	1.97
ST (min/day)	339	233.10	145.03	395	186.13	123.59	344	299.42	150.82	402	250.25	139.65
HRQoL (KIDSCREEN-10 T-Score)	326	43.87	4.19	382	43.15	4.32	342	40.77	4.70	401	40.83	4.27

Note: *N* = number of participants, M = mean, SD = standard deviation, PA = physical activity, ST = screen time, HRQoL = health-related quality of life.

## Data Availability

Original data can be derived from the corresponding author upon request.

## Supplementary Materials

The following are available online at https://www.mdpi.com/2227-9067/8/2/98/s1, Table S1: Differences between study completers and non-completers regarding sociodemographic characteristics and study variables.
