# Supplementary material for: The Impact of COVID-19 on the Interrelation of Physical Activity, Screen Time and Health-Related Quality of Life in Children and Adolescents in Germany: Results of the Motorik-Modul Study
Source: Children (Basel). 2021 Feb 2;8(2):98. doi: 10.3390/children8020098 (PMC7913083; doi:10.3390/children8020098)
Supplement: Supplementary file 1 [file children-08-00098-s001.pdf]

Supplement Table 1. Differences between study completers and non-completers regarding sociodemographic characteristics and study variables.

	Study completers (N = 1,711) Mean(SD) / %	Non-completers (N = 1,132) Mean(SD) / %	P-level	Effect size
Age pre-Covid-19	10.61 (3.87) years	10.36 (4.04) years	0.094	d = 0.06
Gender				
Male	50.2 %	46.0 %	0.049	Φ = 0.04
Female	49.8%	54.0 %		
BMI				
Underweight	10.5 %	8.8 %	<0.001	V = 0.09
Healthy weight	76.8 %	72.4 %		
Overweight	10.3 %	14.0 %		
Obese	2.3 %	4.8 %		
Parental education				
Low	2.9 %	4.3 %	<0.001	V = 0.09
Medium	93.5 %	88.5 %		
High	3.7 %	7.2 %		
PA pre-Covid-19	4.26 (1.79) days	4.09 (1.84) days	0.019	d = 0.11
ST pre-Covid-19	133.22 (123.03) min.	154.73 (126.87) min.	<0.001	d = 0.17
HRQoL pre-Covid-19	44.35 (4.41)	44.10 (4.80)	0.212	d = 0.05

Note: BMI = body-mass-index; PA = physical activity; ST = screen time; min = minutes
